# Reviewed article: Bando DH, Neto FC, Queiroz AP. Stroke mortality negatively associated with health, education, and safety: an ecological study, Minas Gerais, 2014-2022. Epidemiol Serv Saude. 2025:34;e20240820

**DOI:** 10.1590/S2237-96222025v34e20240820.b

**Published:** 2025-09-29

**Authors:** Vinicius Viana Abreu Montanaro

**Affiliations:** 1 SARAH-Brasília, Brasília, DF, Brazil

## Peer review B

## Questionnaire

Does the manuscript contain new and significant information to justify publication? Yes

Does the Abstract (Summary) clearly and accurately describe the content of the article? Yes

Is the problem significant and concisely stated? Yes

Are the methods described comprehensively? Yes

Are the interpretations and conclusions justified by the results? Yes

Is adequate reference made to other work in the field? Yes

## Manuscript Structure

Length of article is: Adequate

Number of tables is: Adequate

Number of figures is: Adequate

Please state any conflict(s) of interest that you have in relation to the review of this paper. None

Interest: Excellent

Quality: Excellent

Originality: Good

Overall: Excellent

Recommendation: Minor Revision

## Comments

O manuscrito é muito interessante e realiza uma análise abrangente sobre a distribuição geográfica e temporal da mortalidade por acidente vascular cerebral (AVC) no estado de Minas Gerais. A metodologia adotada é sólida, com uma análise criteriosa dos dados, e a apresentação e interpretação dos resultados são claras e bem fundamentadas.

Algumas sugestões para aprimorar o manuscrito:

Na seção de Metodologia, os autores mencionam a regressão linear múltipla como sinônimo de regressão espacial (spatial lag). Embora a apresentação nos resultados esteja correta, essa terminologia precisa ser ajustada na metodologia para evitar confusão conceitual.

Seria útil explicar melhor o papel do AIC (Critério de Informação de Akaike), mencionando que ele é utilizado para identificar o modelo que melhor se ajusta aos dados, levando em consideração tanto o ajuste quanto a complexidade do modelo.

Nos resultados, é afirmado que a regressão espacial foi utilizada porque os resíduos da regressão linear apresentaram uma distribuição espacial. Essa explicação deveria ser transferida para a seção de Metodologia, juntamente com a justificativa do uso do parâmetro Rho, para garantir clareza e consistência no texto.

A primeira frase do último parágrafo dos resultados está um pouco confusa. Recomenda-se uma reformulação para melhorar a clareza e a fluidez da ideia apresentada.

Na seção de Limitações, seria relevante discutir as dificuldades em obter dados confiáveis ou de boa qualidade, especialmente em regiões mais distantes e menos favorecidas do estado. Esse ponto é crucial para contextualizar as limitações dos resultados.

